# Glucose determination in human serum by applying inner filter effect quenching mechanism of upconversion nanoparticles

**DOI:** 10.3389/fbioe.2023.1168086

**Published:** 2023-04-10

**Authors:** Xiaojiao Chen, Zhiying Yang, Qiong Chen, Youyu Zhang

**Affiliations:** ^1^ Changsha Health Vocational College, Changsha, China; ^2^ Key Laboratory of Chemical Biology and Traditional Chinese Medicine Research, Ministry of Education, College of Chemistry and Chemical Engineering, Hunan Normal University, Changsha, China

**Keywords:** glucose, upconversion nanoparticles, inner filter effect, 4-amino antipyrine, glucose oxidase

## Abstract

Accurate blood glucose determination is essential to the clinical diagnosis and management of diabetes. This work establishes an inner filter effect (IFE) strategy between upconversion nanoparticles (UCNPs) and quinone-imine complex for glucose monitoring in human serum simply and efficiently. In this system, the enzyme glucose oxidase (GOx) catalyzes the reaction of glucose into hydrogen peroxide (H_2_O_2_) and gluconic acid when compulsion by oxygen. In the presence of horseradish peroxidase (HRP), the produced H_2_O_2_ can catalytically oxidize phenol and 4-amino antipyrine (4-AAP) to generate quinone-imine products. The purple-colored quinone-imine complex effectively absorbed the fluorescence of NaYF_4_:Yb^3+^, Er^3+^ UCNPs, leading to the strong fluorescence quenching of UCNPs through IFE. Thus, a new approach was established for glucose monitoring by determining the fluorescence intensity. Under the optimal condition, this approach shows better linearity to glucose from 2–240 μmol/L with a low detection limit at 1.0 μmol/L. Owing to the excellent fluorescence property and background-free interference of the UCNPs, the biosensor was applied for glucose measurements in human serum and got a satisfactory result. Furthermore, this sensitive and selective biosensor revealed great potential for the quantitative analysis of blood glucose or different kinds of H_2_O_2_-involved biomolecules for the application of clinical diagnosis.

## 1 Introduction

As one of the important biomolecules, glucose is a major source of energy and a significant metabolic intermediate. A healthy person needs to regulate glucose levels in body fluids (Norhammar et al., 2002). When disorder of the carbohydrate metabolism happens, some diseases such as diabetes mellitus and hypoglycemia can occur, which may further increase the risk of heart diseases, kidney diseases, blindness, etc. (Alberti and Zimmet, 2004; Alfadhli, 2018) Currently, the accurate concentrations of blood glucose have been primarily used by clinicians as one of the indicators to make the diagnosis of diabetes and hypoglycemia. In particular, as diabetes is a worldwide public health problem, to the ability to examine the blood glucose level is of great importance. Various approaches to blood glucose testing have been reported including electrochemical methods (Comba et al., 2018; Gopal et al., 2022; Thapa and Heo, 2023; Wang et al., 2023), optical methods (Ramon-Marquez et al., 2017; Kumar and Chauhan, 2022; Zhenglan et al., 2022), and surface plasmon resonance (SPR) spectroscopy (Man et al., 2022; Zhang et al., 2022; Zheng et al., 2022). Although widely applied in commercial glucose tests, electrochemical sensors have easily interfered with biological molecules such as ascorbate (Mani et al., 2015). The potential electrochemical active interferences may result in erroneous sensor responses. Meanwhile, the procedure of SPR is time-consuming because of the tedious probe treatment. Moreover, the experimental cost of SPR is expensive (Zhang et al., 2022). Compared to other techniques, optical methods—particularly fluorescence techniques—have absorbed a lot of interest because of their cost-efficient sensing, the simplicity of the instruments, high sensitivity, and selectivity (Zhang et al., 2023a; Zhu et al., 2023). Most fluorescent sensors for glucose measuring employ conventional organic dyes as well as some organic polymers, and luminescent nanomaterials such as semiconductor quantum dots. However, most of the fluorophores or organic molecules used in previous work have broad emission widths and poor photostability. Their fluorescent signals are easily affected by the decay of chromophores and the working environment (Dong et al., 2013; Khan and Pickup, 2013; Su et al., 2015). To circumvent these issues, kinds of luminescent nanomaterials such as organic-dye doped nanoparticles (Bagheri et al., 2014), semiconductor quantum dots (Chen et al., 2014; Zhai et al., 2016; Samuei et al., 2017), fluorescent carbon nanodots (Ma et al., 2017; Zou et al., 2018), and metal nanoclusters (Wang et al., 2014; Cheng et al., 2018) have been devised as the analytical probe because of their preferable photoluminescence properties. Unfortunately, these fluorescence nanostructures have inherent disadvantages, namely shorter wavelength excitation, auto-fluorescence from the background, and possible damage to biological samples (Hardman, 2006; Roberts et al., 2013). Therefore, it is of utmost importance to use excellent materials that can make up for these deficiencies.

As a better choice, upconversion nanoparticles (UCNPs) can transform low-energy near-infrared (NIR) emissions to high-energy visible emissions with large stokes shifts (Dong et al., 2012a). In comparison with visible light excitation, the specific NIR excitation of UCNPs allows weaker autofluorescence interference and lower phototoxicity (Wang et al., 2013; Yang et al., 2013). Apart from the superior photon upconversion properties, rare-earth doped UCNPs display stability against photobleaching, sharp emission bandwidth, and a long lifetime, which render them specifically useful for the construction of fluorescence biosensors (Wang et al., 2011; Zhang et al., 2015; Pu et al., 2018). The mechanism of the inner filter effect (IFE) is an effective strategy by transforming the absorption response to a fluorescence signal, which opens up new frontiers in converting conventional colorimetric protocol into fluorescent sensing (Zhang et al., 2009; Kong et al., 2016; Chen et al., 2017; Sun et al., 2018). For example, Ruiting Zhang uses carbon quantum dots nano-fluorescence probe for rapid and sensitive detection of methyl parathion in rice based on the inner filter effect (Zhang et al., 2023b). It is reported that the energy conversion model of IFE considerably enhanced the detecting sensitivity and reduced the detection limit of the analyte relative to the absorbance alone (Chen et al., 2018). It supplies some feasibility for developing a typical IFE-based fluorescence biosensor with good repeatability and less disturbance.

**SCHEME 1:**
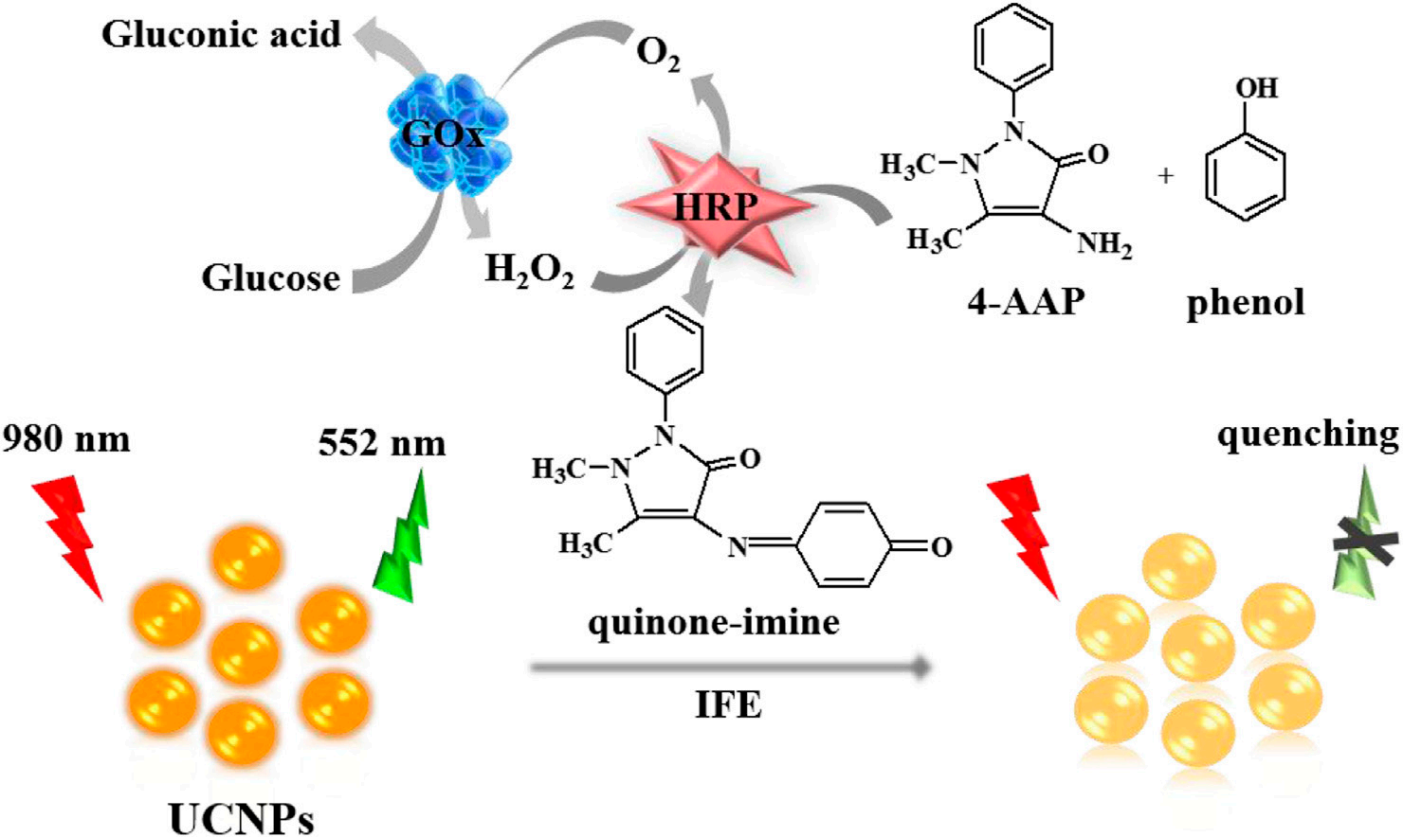
Schematic illustration of the glucose detection mechanism using the UCNPs.

Herein, we constructed a facile, label-free sensing platform for hydrogen peroxide (H_2_O_2_) and glucose tests basing the quench of NaYF_4_:Yb^3+^, Er^3+^ UCNPs fluorescence by IFE. This implementation plan is displayed in Scheme 1. Firstly, the enzyme glucose oxidase (GOx) catalyst the conversion of glucose and oxygen into gluconic acid and H_2_O_2_. The produced H_2_O_2_ then reacts with 4-amino antipyrine (4-AAP) and phenol to generate a quinone-imine complex when horseradish peroxidase (HRP) exists. Consequently, the purple-colored quinone-imine product effectively absorbs the fluorescence of UCNPs, which causes a distinct quench of the UCNPs’ fluorescence. The quenching efficiency is proportionate to the increased amount of glucose. Thus, this biosensor ensures the convenient measurement of glucose.

## 2 Experimental

### 2.1 Materials and apparatus

Erbium oxide (Er_2_O_3_, 99.99%), ytterbium oxide (Yb_2_O_3_, 99.99%), and yttrium oxide (Y_2_O_3_, 99.99%) bought in Sinopharm Chemical Reagent Co., Ltd. (Shanghai, China). Their nitrate solution was achieved dissolution in hot nitric acid and then dissolved in deionized water to respectively obtain 0.1 M, 0.1 M, and 0.1 M eventual concentrations. Sodium citrate, cetyltrimethylammonium bromide (CTAB), sodium fluoride (NaF), glucose, HRP, 4-AAP, and the rest sugars purchased in Sigma (Shanghai, China). GOx was acquired from Sigma-Aldrich (Shanghai, China). Phenol, H_2_O_2_, and nitric acid (HNO_3_) were purchased in Alfa Aesar (Shanghai, China). Other reagents mentioned in the article were from Aladdin (Shanghai, China). The detection buffer was pH 7.0 PBS of 100 mM. All chemicals were analytically pure and used Millipore Milli-Q ultrapure water during all experiments. Human serum samples were provided by the Hospital of Hunan Normal University, China.

The UCNPs’ fluorescence signal has recorded by an F-4500 fluorescence spectrophotometer (Hitachi Ltd., Japan), which used the external 980 nm laser as excitation light. Transmission electron microscopy (TEM, JEOL-1230, Japan) was applied for the size and character morphology describing of UCNPs. The UV-vis absorption spectra were collected on a UV-2450 spectrophotometer (Shimadzu Co., Japan). The Fourier transform infrared (FT-IR) spectra were conducted on a Nicolet Nexus 670 FT-IR spectrometer (Nicolet Instrument Co., USA). The crystalline phases of UCNPs were measured by a Rigaku 2,500 (Japan) X-ray diffractometer (XRD).

### 2.2 Synthesis of UCNPs

The NaYF_4_:Yb^3+^, Er^3+^ UCNPs were prepared based on a solvothermal synthesis technique (Chen et al., 2012; Ye et al., 2014). In short, the mixing of 0.2 mL of 0.1 M Er(NO_3_)_3_, 1.0 mL of 0.1 M Yb(NO_3_)_3_, and 8.8 mL of 0.1 M Y(NO_3_)_3_ was stirred together with 7.5 mL of 0.1 M sodium citrate to prepare a white metal-citrate compound. After that, add 25 mL ethanol and 0.2 g CTAB to the mixture, keeping 15 min stirring. Then dropwise add 12 mL of 1 M NaF to the sample, while continuing mixing with 1 hour of vigorous stirring. Subsequently, add 2 mL HNO_3_ into the solution and transferred the whole mixture into a Teflon-lined autoclave, keeping heating at 180°C for 4 h. And then, remove the final sample solution, cool down, and centrifugate. Finally, the obtained precipitates were sequentially cleaned with deionized water and ethanol 3 times. Vacuum-dried before use.

During the experiment, the NaYF_4_:Yb^3+^, Er^3+^ UCNPs were uniformly dispersed in PBS buffer solution to obtain a 0.1 mg/mL solution, which was then used in the fluorescence analysis.

### 2.3 Detection of H_2_O_2_

Firstly, different concentrations of H_2_O_2_ were mixed with the complex containing 50 μL UCNPs (0.1 mg/mL), 5 μL HRP (25 μg/mL), 15 μL 4-APP (25 mM), and 40 μL phenol (25 mM). Then dilute it well with a total of 500 μL after a 4 min reaction at room temperature (RT). Followed by detecting the fluorescence emission spectra of the resultant products. All experiments were measured at RT (25 ± 1.0°C).

### 2.4 Glucose determination

Initially, various amounts of glucose were added into a 20 μL of pH7.0 PBS buffer of 100 mM containing 10 μL GOx (1 mg/mL). After interaction at 37°C for 40 min, the solution was mixed with 15 μL HRP (25 μg/mL), 15 μL 4-APP (25 mM), 40 μL phenol (25 mM), and 50 μL UCNPs (0.1 mg/mL) for 4 min. And then, diluted the mixture to a total of 500 μL, and recorded the fluorescence emission spectra of the final product.

### 2.5 Glucose determination in human serum samples

Before the measurement, the original human serum samples have diluted with pH7.4 PBS buffer of 100 mM to 100 times. Briefly, the definite glucose levels in human serum samples were tested by the proposed sensor first. After that spiking accurate amount of glucose to the serum samples for further analysis.

## 3 Results and discussion

### 3.1 Characterization of the UCNPs

**FIGURE 1:**
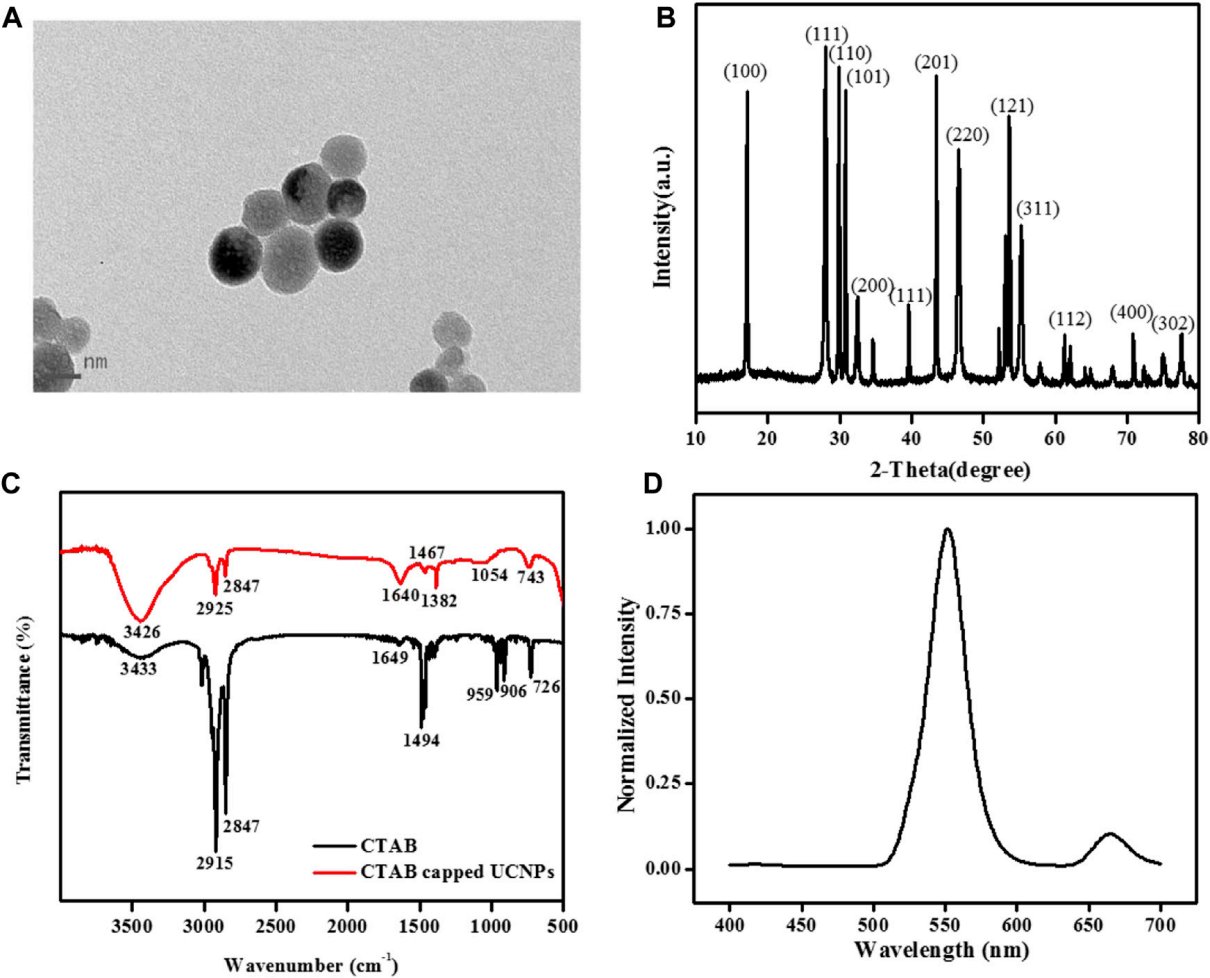
**(A)** TEM images, **(B)** XRD pattern, and **(C)** FT-IR spectra of NaYF_4_:Yb^3+^, Er^3+^ UCNPs. **(D)** The normalized fluorescence spectrum of the UCNPs solution under 980 nm laser excitation. [UCNPs]: 0.1 mg/mL.

Firstly, TEM, XRD, FT-IR, and fluorescence spectrums were used to represent the morphology character and the optical properties of UCNPs (Figure 1). As seen in Figure 1A, the TEM images of the UCNPs exhibit good dispersivity in water with about 34 nm mean diameter. Figure 1B shows the classic XRD patterns of the UCNPs. The prepared UCNPs’ whole diffraction peaks are consistent with the normative cubic UCNPs crystal (JCPDS no.77-2042), demonstrating the successful obtain of the synthesized NaYF_4_:Yb^3+^, Er^3+^ nanocrystals. Moreover, Figure 1C of the FT-IR spectra presented the UCNPs’ functional groups on the surface. The 2,915 cm^-1^ and 2,847 cm^-1^ absorptions belong to the asymmetric and symmetric stretching vibration of C-H (-CH_3_ and -CH_2_-), and the 1,494 cm^-1^ peak ascribed to the typical amine groups (-NH_2_) peak, exhibiting that the UCNPs surface has functionalized with CTAB. Owing to the surface of nanoparticles being covered with CTAB, the nanomaterials are stable and have favorable dispersibility in an aqueous solution. For further study, it found that the UCNPs fluorescence signal essentially stay the same within 60 min (Supplementary Figure S1A) and was not influenced by pH (Supplementary Figure S1B). When excited by 980 nm diode laser, the fluorescent spectra of NaYF_4_:Yb^3+^, Er^3+^ UCNPs show a strong characteristic green emission band at 552 nm and a red emission band at 665 nm, belonging to transitions from the ^4^S_3/2_ and ^4^F_9/2_ excited states to the ^4^I_15/2_ ground state of the Er^3+^ ions, respectively (Figure 1D) (Yin et al., 2014).

### 3.2 Exploration of the fluorescence quenching principle

**FIGURE 2:**
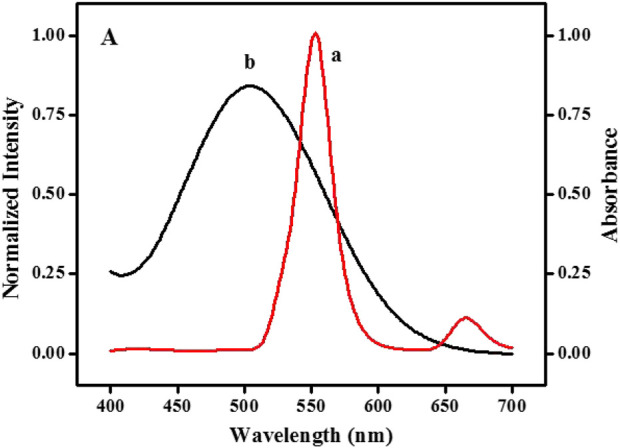
Spectral overlap: emission spectrum of NaYF_4_:Yb^3+^, Er^3+^ UCNPs (curve a) and absorption spectrum of the quinone-imine system (curve b).

The fluorescence quenching principle of UCNPs may relate to IFE or fluorescence resonance energy transfer (FRET) (Chen et al., 2017). UV-vis absorption spectroscopy and fluorescence emission spectra were used for further investigation. Supplementary Figure S2 illustrates that whether UCNPs exist, the absorption bands’ intensity and position of quinone-imine products do not change (curve f and curve g). The results stated that there is no complex forming and FRET between UCNPs and quinone-imine is impossible. According to the report, the fluorescence intensity of fluorophores can be effectively adjusted by absorbers by utilizing IFE only when there has considerable overlap between the absorption band of the absorber and the excitation and/or emission bands of the fluorophores (Dong et al., 2012b; Zheng et al., 2013). Figure 2 shows that the typical fluorescence emission of UCNPs appeared at 552 nm (curve a), and the maximum absorption band of quinone-imine was located at 502 nm (curve b). The fluorescence spectra of UCNPs significantly overlap with the absorption spectra of quinone-imine in 500–650 nm, which ensures that an effective IFE could happen between them. Judging from the above analysis, the fluorescence decrease of UCNPs by quinone-imine should be attributed to IFE.

### 3.3 Feasibility study of the analysis

**FIGURE 3:**
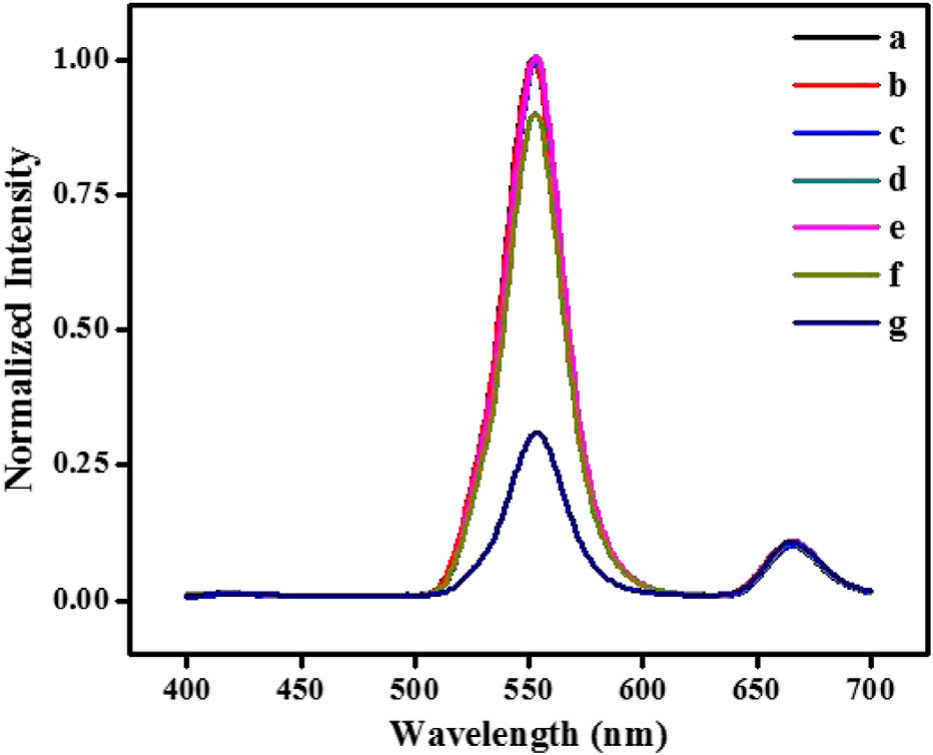
The fluorescence spectra of NaYF_4_:Yb^3+^, Er^3+^ UCNPs **(A)** in the absence and presence of GOx+Glu+HRP+4-AAP **(B)**, GOx+Glu+HRP+phenol **(C)**, GOx+Glu+phenol+4-AAP **(D)**, GOx+HRP+phenol+4-AAP **(E)**, Glu+HRP+phenol+4-AAP **(F)** and GOx+Glu+HRP+phenol+4-AAP **(G)**, respectively. [UCNPs]: 0.1 mg/mL, [GOx]: 20 μg/mL, [Glu]: 240 μmol/L, [HRP]: 0.75 μg/mL, [4-AAP]: 0.75 mmol/L, [phenol]: 2.0 mmol/L.

The strategy of glucose detection by UCNPs is based on GOx and HRP-catalyzed reactions. First of all, The GOx catalyzes glucose to create H_2_O_2_ and gluconic acid while oxygen exists. In the presence of HRP, the generated H_2_O_2_ can catalytically oxidize 4-AAP and phenol to form quinone-imine products. The purple-colored quinone-imine significantly absorbs the fluorescence of nanoparticles, and the signal of UCNPs fluorescence efficiently decreased. It can see in Figure 3 that the representative fluorescence peak of UCNPs is centered at 552 nm and 665 nm (curve a). When UCNPs were mixed with the integrated GOx-glucose-HRP-phenol-4-AAP system, the fluorescent signal of UCNPs at 552 nm was visibly quenched, and the emission peak at 665 nm was kept unchanged (curve g). To study the affection of related factors, it tests the UCNPs’ fluorescence spectra while glucose, GOx, HRP, 4-AAP, and phenol exists, respectively. The emission signal of GOx+Glu+HRP+4-AAP (b), GOx+Glu+HRP+phenol (c), GOx+Glu+phenol+4-AAP (d), GOx+HRP+phenol+4-AAP (e) and Glu+HRP+phenol+4-AAP (f) has no significant change with UCNPs. Therefore, the results exhibited that the sensing of glucose is feasible.

### 3.4 Optimization of the analyzed parameters

**FIGURE 4:**
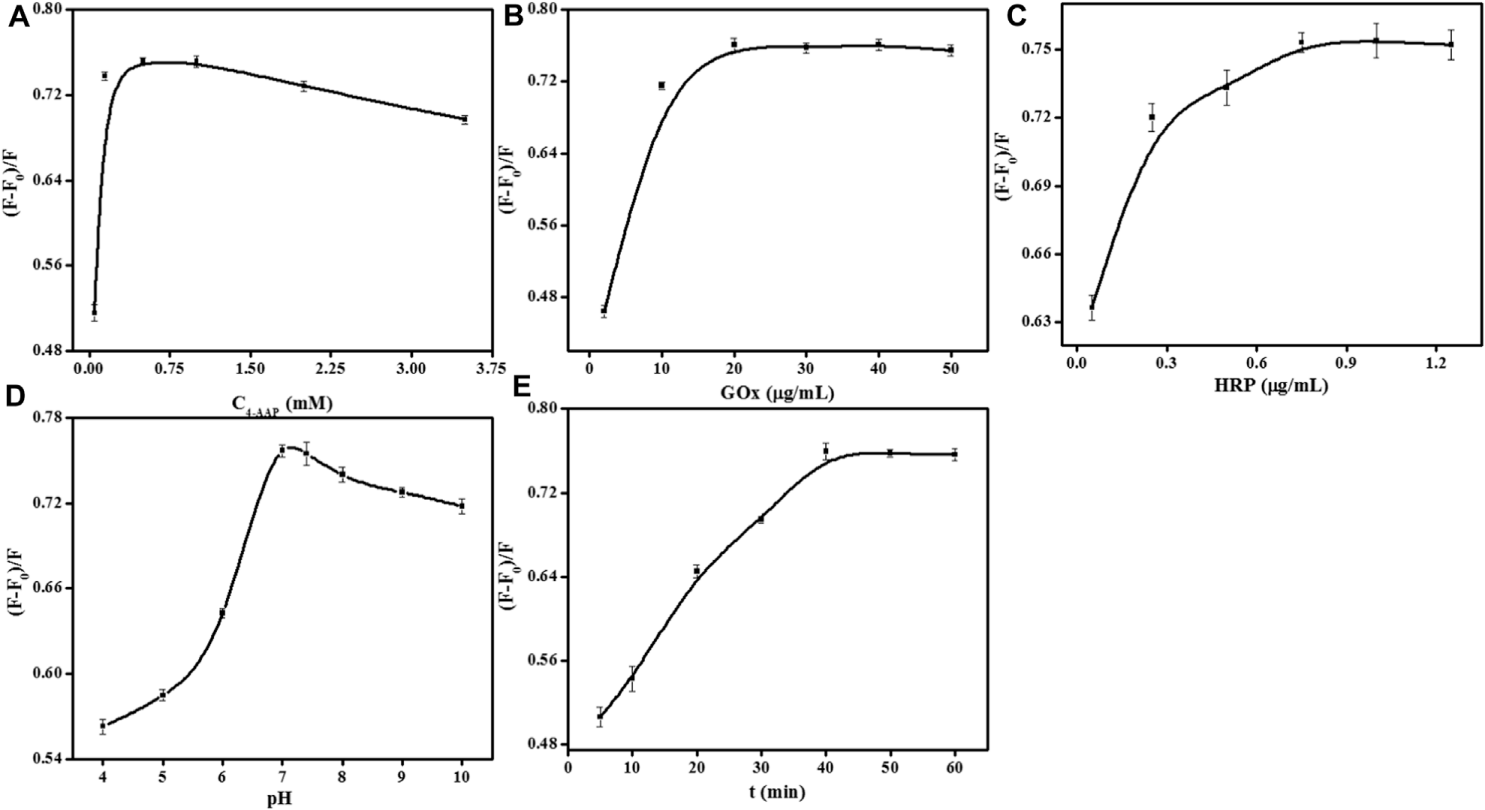
Effects of 4-AAP concentration **(A)**, GOx concentration **(B)**, HRP concentration **(C)**, pH **(D)**, and incubation time **(E)** on the fluorescence responses of the sensor for glucose detection. [UCNPs]: 0.1 mg/mL, [GOx]: 20 μg/mL, [Glu]: 240 μmol/L, [HRP]: 0.75 μg/mL, [4-AAP]: 0.75 mmol/L, [phenol]: 2.0 mmol/L.

The detection parameters such as the concentration of 4-AAP, Gox, and HRP, media pH, and incubation time were evaluated to ensure perfect sensitivity. For selecting the most appropriate experimental conditions, the quenching efficiency of the fluorescence signal is employed as a standard in this work. The quenching efficiency of the fluorescence signal is defined as (F-F_0_)/F, while F and F_0_ refer to the 552 nm fluorescence intensity respectively in the absence and presence of glucose. The concentration of 4-AAP was optimized initially (Figure 4A). As shown that the fluorescence quenching efficiency appeared maximal when 0.75 mM 4-AAP was added to the detection solution. Thus, 0.75 mM of 4-AAP was used for glucose assay. To avoid the waste of enzymes, we also investigated the concentration of GOx and HRP. In Figure 4B, the fluorescence quenching efficiency rapidly increased with the amounts of GOx and stabilized when kept at 20 ug/mL. So, we select 20 ug/mL of GOx for further experiment. Meanwhile, increasing fluorescence quenching efficiency was observed with the gradual addition of HRP and stabilized at 0.75 ug/mL (Figure 4C). Hence, 0.75 ug/mL of HRP was chosen as the feasible concentration used throughout this study. Although pH value does not influence the fluorescent intensity of UCNPs, the activities of GOx and HRP are highly susceptible to pH. Therefore, there is essential to detect the reaction pH for the best enzyme activity. Figure 4D displayed the pH affection from 4.0 to 10.0 on the fluorescence quenching efficiency. In Figure 4D, when the pH value reaches 8.0–10.0, it is a strong alkaline condition, while both GOx and HRP will gradually be inactivated under strongly alkaline conditions. The fluorescence quenching increased to the maximum in pH 7.0 PBS solution (100 mM), confirming that the most suitable pH was around 7.0. In addition, the incubation time can also affect the sensor signal. The UCNPs’ fluorescence quenching gradually increased with the incubation time and was kept balanced at 40 min (Figure 4E). Thus, the best incubation time was 40 min to obtain a suitable signal.

### 3.5 Quantitative analysis of H_2_O_2_

**FIGURE 5:**
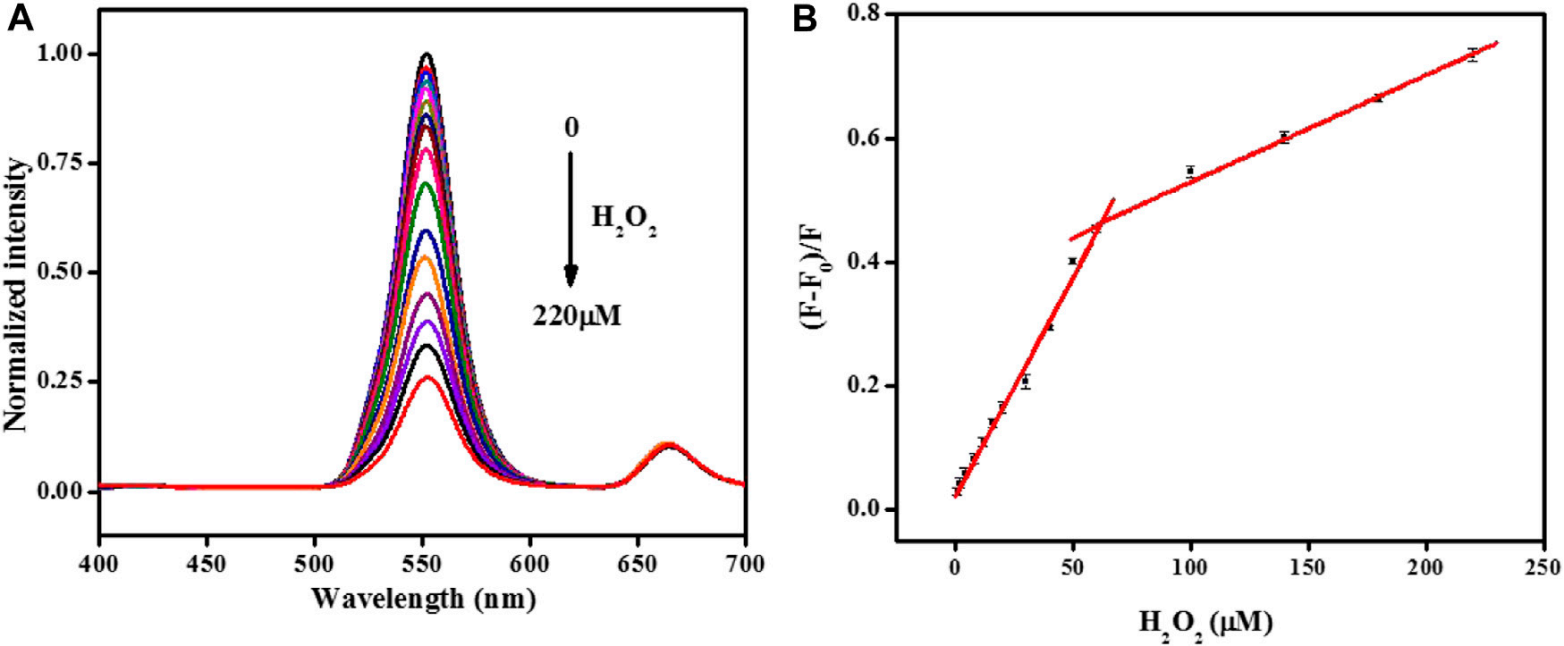
**(A)**The normalized fluorescence emission spectra of the UCNPs in the presence of different concentrations of H_2_O_2_ (from 0 to 220: 0, 1, 2, 4, 8, 12, 16, 20, 30, 40, 50, 60, 100, 140, 180, and 220 μmol/L). **(B)** Relationship between the fluorescence quenching efficiency and the concentration of H_2_O_2_. [UCNPs]: 0.1 mg/mL, [HRP]: 0.75 μg/mL, [4-AAP]: 0.75 mmol/L, [phenol]: 2.0 mmol/L.

Based on the most suitable detection conditions, the possibility of quantitative monitoring of H_2_O_2_ was investigated. Figure 5A displays that the standard fluorescent signal of UCNPs gradually decreased with H_2_O_2_ ranging from 1–220 μM. Taking the concentration of H_2_O_2_ as the *X*-axis, and the fluorescence quenching efficiency as the *Y*-axis, draw the standard curve in Figure 5B. The corresponding equation of linear regression for the amount of 1–60 μM was (F-F_0_)/F = 0.0071C + 0.021 (*R* ^2^ = 0.991), and (F-F_0_)/F = 0.0017C + 0.356 (*R* ^2^ = 0.993) in the range of 60–220 μM. The detection limit of 0.15 μM (S/N = 3) was achieved, demonstrating that the evolution of fluorescence change is optimal for H_2_O_2_ analysis, as shown in Supplementary Figure S1 and Supplementary Figure S2.

### 3.6 Quantitative analysis of glucose

**FIGURE 6:**
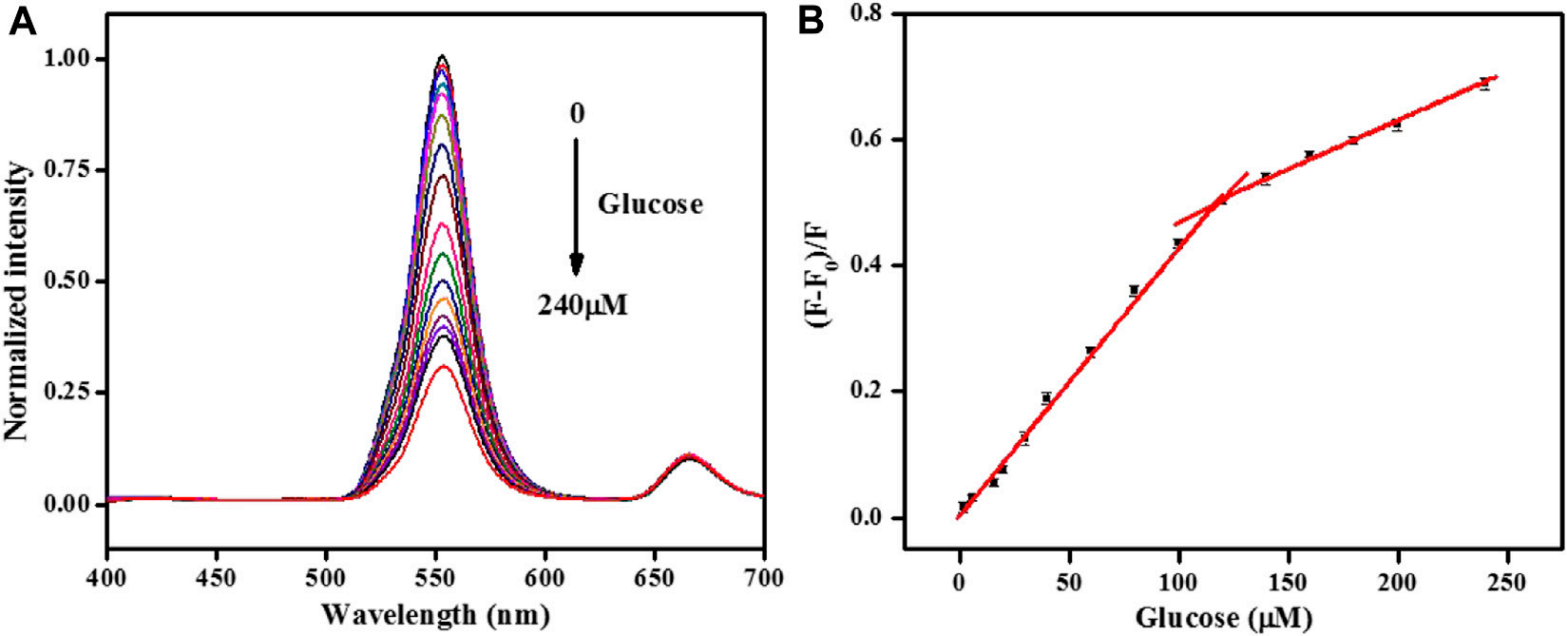
**(A)**The normalized fluorescence emission spectra of the UCNPs in the presence of different concentrations of glucose (from 0 to 240: 0, 2, 6, 16, 20, 30, 40, 60, 80, 100, 120, 140, 160, 180, 200, and 240 μmol/L). **(B)** Relationship between the fluorescence quenching efficiency and the concentration of glucose. [UCNPs]: 0.1 mg/mL, [GOx]: 20 μg/mL, [HRP]: 0.75 μg/mL, [4-AAP]: 0.75 mmol/L, [phenol]: 2.0 mmol/L.

Under optimum parameters, the analytical performance for glucose detection was measured. Figure 6A shows that the normalized fluorescence peak at 552 nm of the UCNPs quenched gradually with the addition of glucose. Good linearity has been achieved by plotting (F-F_0_)/F *versus* the amount of glucose ranging from 2–120 μmol/L and 120–240 μmol/L with the equations below: (F-F_0_)/F = 0.0043C + 0.003 and (F-F_0_)/F = 0.0016C + 0.321, and their correlation coefficient were 0.997 and 0.987, respectively. The detection limit was 1.0 μM (S/N = 3). More importantly, this sensor exhibited a lower detection limit than some reported fluorescence methods for glucose (Supplementary Table S1) (Lu et al., 2021; Fu et al., 2022; Gao et al., 2023; Zha et al., 2023). Such a low limit of detection stated that the proposed sensing strategy performed well with low background signal by NIR excitation.

### 3.7 Selectivity of the proposed biosensor

**FIGURE 7:**
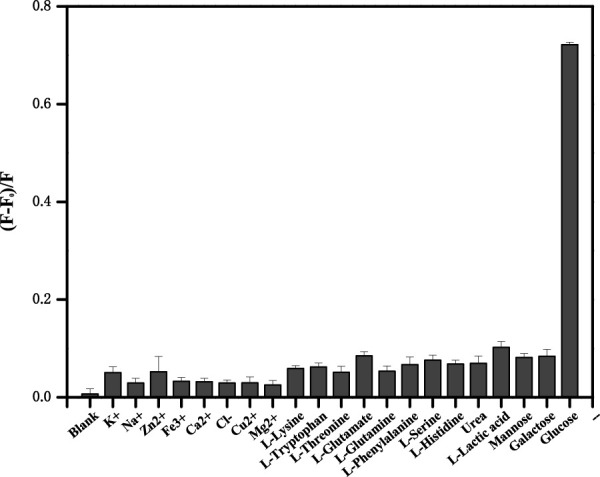
The upconversion fluorescence response in the presence of different metal ions, some amino acids, small biological molecules (2.4 mmol/L each), and saccharides (0.24 mmol/L each). Error bars represent the standard deviations of three repetitive experiments.

The investigation of selectivity is of great importance for this sensing platform. Therefore, the effects of some potentially ordinary interfering substances including mental ions (K^+^, Na^+,^ and Zn^2+^), some amino acids (lysine, tryptophan, threonine, glutamate, glutamine, phenylalanine, serine, and histidine), small biological molecules (urea and lactic acid) and saccharides (mannose and galactose) were tested. The platform can detect the impact of different kinds of interfering species on the fluorescence quenching efficiency (Figure 7). It is clear that most of these chemicals have slight signals and even reach high concentrations. The specific selectivity could ascribe to the high affinity between GOx and glucose. The results proved that this biosensor showed pronounced selection toward glucose analysis.

### 3.8 Determination of practical samples

For evaluating the practical application of this proposed assay, we use the biosensor to test the glucose level in human serum samples. Consideration of the glucose concentration in a healthy human serum environment and the range of linearity of this sensing system, diluting the human serum samples 100 times before experiments. Table 1 lists the results carried out by the standard addition method. We attained 98.5%–101.5% good recoveries with less than 3.45% relative standard deviation (RSD, n = 3), illustrating that the sensitive fluorescence biosensor can be applied to glucose testing in practical serum samples with satisfactory results.

**TABLE 1:** The application of the method for the determination of serum samples with different amounts of glucose.

Sample	Added (μmol/L)	Found (μmol/L)	Recovery (%)	RSD (*n* = 3,%)
1	0.00	54.58	—	2.9
2	30.00	85.86	101.5	3.7
3	70.00	123.15	98.9	3.0
4	100.00	152.26	98.5	3.5

## 4 Conclusion

In summary, we constructed a facile fluorescent sensing platform for the analysis of H_2_O_2_ and glucose basing the fluorescence quenching of UCNPs. Compared with previous research for glucose determination, the application of this proposed biosensor shows its superiority. Initially, the chromogenic reaction of H_2_O_2_-phenol-4-AAP systems turned into IFE-based fluorescent sensing in a simple turn-off mode, which enhanced the analyzing properties and the detection sensitivity. Secondly, negligible background interference and lower detection limit can be obtained by benefiting from the unique excitation and emission properties of UCNPs. Lastly, with no special surface modification of UCNPs nor a complicated probe fabrication procedure, the label-free sensor undoubtedly provides considerable flexibility for the assay. Therefore, this biosensor ensures accurate, convenient, and efficient measurement of glucose in human serum.

The fluorescence approach provided some remarkable advantages in selectively, simplicity, low price, and high sensitivity, which make it capable of applying to the detection of glucose levels in human serum. Meanwhile, this measurement method can be readily expanded to diagnose other different H_2_O_2_-involved biomolecules like triglyceride, uric acid, and cholesterol in the field of clinical bioassays. Therefore, we believe that the method may offer a promising tool for exploiting convenient, simple, and low-cost sensors for biochemical and clinical applications, for instance, *in-vitro* determination of serum biomarkers for health management and disease diagnosis.

## Data Availability

The original contributions presented in the study are included in the article/Supplementary Material, further inquiries can be directed to the corresponding author.

## Supplementary material

Supplementary Figure S1 Effects of time **(A)** and pH **(B)** on the fluorescence responses of the UCNPs. [UCNPs]: 0.1 mg/mL.

Supplementary Figure S2 The absorbance spectra of Glu+HRP+phenol+4-AAP **(A)**, GOx+HRP+phenol+4-AAP **(B)**, GOx+Glu+HRP+phenol **(C)**, GOx+Glu+HRP+4-AAP **(D)**, GOx+Glu+phenol+4-AAP **(E)**, and GOx+Glu+HRP+phenol+4-AAP system in the absence **(F)** and presence **(G)** of NaYF4:Yb3+, Er3+ UCNPs. [UCNPs]: 0.1 mg/mL, [GOx]: 20 μg/mL, [Glu]: 240 μmol/L, [HRP]: 0.75 μg/mL, [4-AAP]: 0.75 mmol/L, [phenol]: 2.0 mmol/L.

Supplementary Table S1 Comparison of different fluorescence methods for the determination of glucose.
