# Supplementary material for: Glucose determination in human serum by applying inner filter effect quenching mechanism of upconversion nanoparticles
Source: Front Bioeng Biotechnol. 2023 Apr 10;11:1168086. doi: 10.3389/fbioe.2023.1168086 (PMC10123268; doi:10.3389/fbioe.2023.1168086)
Supplement: Supplementary file 1 [file DataSheet1.docx]

Supporting Information

Glucose Determination in [Human Serum](javascript:;) by Applying Inner Filter Effect Quenching Mechanism of Upconversion Nanoparticles

Xiaojiao Chen^1^, Zhiying Yang^1^, Qiong Chen^1^ and Youyu Zhang ^2^*

^1^*(**Changsha Health Vocational College, Changsha 410100, China)* ^2^*(Key Laboratory of Chemical Biology and Traditional Chinese Medicine Research (Ministry of Education),College of Chemistry and Chemical Engineering, Hunan Normal University, Changsha 410081, China)*

* Corresponding author: Tel: +86-731-88865515; fax: +86-731-88865515;

E-mail address: zhangyy@ hunnu.edu.cn


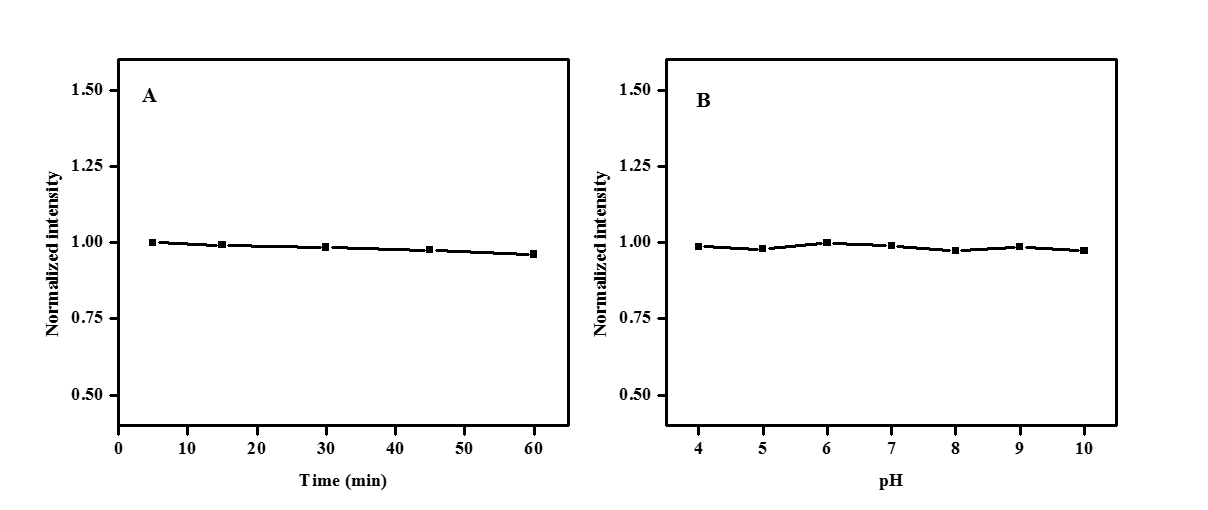


Fig.S1 Effects of time(A) and pH (B) on the fluorescence responses of the UCNPs. [UCNPs]: 0.1 mg/mL


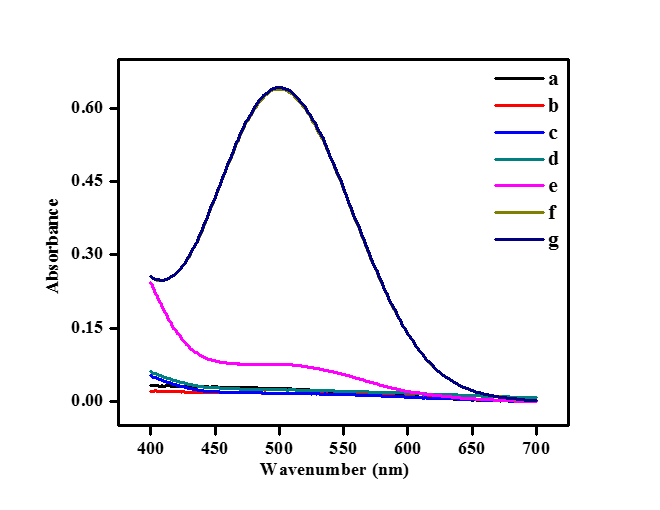


Fig.S2 The absorbance spectra of Glu+HRP+phenol+4-AAP (a), GOx+HRP+phenol+4-AAP (b), GOx+Glu+HRP+phenol (c), GOx+Glu+HRP+4-AAP (d), GOx+Glu+phenol+4-AAP (e), and GOx+Glu+HRP+phenol+4-AAP system in the absence (f) and presence (g) of NaYF_4_:Yb^3+^, Er^3+^ UCNPs. [UCNPs]: 0.1 mg/mL, [GOx]: 20 μg/mL, [Glu]: 240 μmol/L, [HRP]: 0.75 μg/mL, [4-AAP]: 0.75 mmol/L, [phenol]: 2.0 mmol/L.

TableS1 Comparison of different fluorescence methods for the determination of glucose.

| **Methods** | **System and materials** | **Linear range(μmol/L)** | **The detection limit (μmol/L)** | **Reference** |
| --- | --- | --- | --- | --- |
| Ratiometric fluorescence, nanozyme-based | copper-doped carbon-based nanozyme and Mg/N doped carbon quantum dots | 2-400 | 1.56 | [[1](#_ENREF_1)] |
| Fluorescence, turn-on detection | the NaGdF_4_: Yb^3+^, Er^3+^ cores, and Ag layers upconversion nanoprobes | 0-3.2 | 1.77 | [[2](#_ENREF_2)] |
| Limitation-induced fluorescence enhancement | carbon nanoparticles | 50-2000 | 10 | [[3](#_ENREF_3)] |
| Fluorescence, glucose, and pH-responsive | silicon-coated perovskite quantum dots, CsPbBr_3_@SiO_2_ | 45-480 | 18.5 | [[4](#_ENREF_4)] |
| Fluorescence, inner filter effect | NaYF_4_:Yb^3+^, Er^3+^ upconversion nanoparticles | 2-240 | 1.0 | This work |

**References**

[1] Q Fu, X Zhou, M Wang, and X Su, Nanozyme-based sensitive ratiometric fluorescence detection platform for glucose, Analytica Chimica Acta, 1216 (2022) 339993-339996.

[2] S Zha, H Li, G.L Law, K.L Wong, and A.H, Sensitive and responsive upconversion nanoprobes for fluorescence turn-on detection of glucose concentration, Materials & Design, 227 (2023) 111800-111805.

[3] Q Lu, T Huang, J Zhou, Y Zeng, C Wu, M Liu, H Li, Y Zhang, and S Yao, Limitation-induced fluorescence enhancement of carbon nanoparticles and their application for glucose detection, Spectrochimica Acta Part A: Molecular and Biomolecular Spectroscopy, 244 (2021) 118893-118897.

[4] M Gao, J Li, L Qiu, X Xia, X Cheng, F Xu, and G Xu, Glucose and pH responsive fluorescence detection system based on simple synthesis of silicon-coated perovskite quantum dots, Spectrochimica Acta Part A: Molecular and Biomolecular Spectroscopy, 289 (2023) 122212-122216.
